# The Treatment of Acute Summer Diarrhœa and Vomiting of Infants

**Published:** 1907-08-03

**Authors:** G. E. Halstead

**Affiliations:** Consulting Surgeon to Ramsgate and St. Lawrence Royal Dispensary


### SPECIAL ARTICLE.

### THE TREATMENT OF ACUTE SUMMER DIARRHŒA AND VOMITING OF INFANTS.

By G. E. HALSTEAD, M.D., Consulting Surgeon to Ramsgate and St. Lawrence Royal Dispensary.

The following article embodies views which I first expressed in 1897. It is very possible that the methods I advocate are now more generally adopted than they were then, but with the approach of the summer season I may perhaps repeat my views upon the treatment of acute summer diarrhœa and vomiting of infants, in the hope that they may be of some service to at any rate the young practitioner.

The present paper is concerned with the treatment by diet and drugs of acute infantile diarrhœa during the first week of the illness. On some few points concerning this disease medical men are agreed. All acknowledge its fatality and its epidemic character. All seem agreed, also, that a certain percentage of the cases are apparently hopeless from the first, and that in them all our efforts are in vain. It is still more certain that the majority of cases get well under very various, and even almost opposite, methods of treatment, so perhaps one may surmise that they would have recovered without any special medicinal treatment at all. Here, however, unanimity in the profession may be said almost to cease.

The present writer lives at the seaside, and has had considerable experience in these cases, for it seems as if almost all the babies in England are centrifugalised to the edge of the land in August. Whatever weight may attach to the numerous theories about the etiology of summer diarrhœa, there can be no doubt as to the bad effect which the heat and dust of August have on infants' food. From the cow's udder to the baby's mouth milk has a dangerous journey—dirty cow, milk-pail, milk-can, railway transport, street dairy, milkman's can, milk jug with dust blown on to it from the open window of the cellar kitchen, feeding-bottle with its lengthy and imperfectly cleaned tube—surely it is not assuming much to guess that in most cases, if not in all, a dose of germ-laden or partly sour milk has started the trouble. Of course, in many cases food evidently improper at the baby's age, even if its quality be good, has been given, such as porridge to a tiny baby and bites of cherry or plum to one a year old. When a baby has diarrhœa and the cause is in question, "cherchez la nourriture." In August, at the seaside, mothers and nurses fancy that babies can digest anything (especially if the feet be dipped into the sea). "He seemed to like it" is considered sufficient justification for all such experiments.

As the purpose of this paper is treatment of disease rather than its description, we need not enter much into the distinctions between the varieties of diarrhœa. Three chief kinds are noticeable:

1. Simple (mild).
2. Inflammatory (stools often bloody).
3. Choleraic (septic).

But the simple may become inflammatory or choleraic; the inflammatory and the choleraic may be combined; the inflammatory may become choleraic; or if the shock of a choleraic attack be survived, an inflammatory attack may ensue.

However, so far as the diagnosis of simple, inflammatory, or choleraic diarrhœa is to bear on our treatment, the following points may be noted:—

1. If we think we have a simple mild attack to deal with, we had better treat it as carefully as if worse symptoms were to follow on in a day or two.

2. If we think the attack is inflammatory, the temperature (taken preferably in the rectum) should be carefully watched, so that it may be kept within bounds by tepid sponging. It is probably in the cases which present bloody motions that ipecacuanha has done good.

3. If from the sunken state of the baby we consider the attack to be one of choleraic diarrhœa, then the condition and treatment of the baby is for the time of more importance than the condition and treatment of the baby's bowels; shock, collapse, prostration are the dangers. The nervous system is poisoned, the vascular system is partly emptied, and life may be a question of hours. Into the treatment of this condition of collapse it is not the writer's purpose to enter fully, for there is hardly any divergence of opinion about it. Some practitioners are more energetic in their efforts than others, but the ideas are the same, and find vent in the following list of agents: Stimulants, such as brandy, up to ʒj. in twenty-four hours, by mouth, by rectum, and hypodermically; hot baths, with or without a tablespoonful of mustard; cotton-wool pads and flannel clothing; hot water bottles near the feet and in the bed; rectal injections of warm saline solution, as much at a time and as often as can be retained, *e.g.*, ℥iv. at a time of common salt solution (ʒj.—Oj.); strychnine injections hypodermically gr. $\frac{1}{400}$ every hour; hourly morphine injections hypodermically gr. $\frac{1}{100}$, combined with atropine gr. $\frac{1}{800}$. Morphine is said by some authorities to be the best remedy for collapse; it may be so. As to the safety and value of strychnine and morphine in this condition, the present writer is not competent to judge, but he is competent to advise a young practitioner who is inclined to inject these strong remedies into tiny babies balanced between life and death, to do so only after dividing the responsibility with another medical man. If the child is not already in a state of stupor, it soon may be; the hypodermic injection and its donor may be blamed for this. The writer has not the slightest doubt that the wisest course to adopt in the treatment of all cases is to assume that something taken in by the mouth has caused the trouble; and therefore his first step is to give an aperient to remove all irritating matter. To warrant the use of an aperient it is not necessary that there should be present in the bowel such things as a piece of pear or a decomposing shrimp; when once the bowels are upset in their process of normal digestion, even good food, such as sterilised milk, will be only partly digested, and will then undergo some process of abnormal decomposition. The sooner one can remove by an aperient this effete food with its contained bacteria and their poisonous products the better for the patient.

We begin the treatment of the bowels therefore by an aperient selected, say, from the following list: Castor oil ʒj., tinct. rhei ♏xv., calomel gr. j., hyd. cum. cret. gr. iij. If the child has been recently vomiting, wait two hours since the last vomit before administering the aperient or anything else at all by the mouth. Even the remedies for collapse must be rectal or hypodermic. Vomiting must be stopped for two reasons:—

1. It is most exhausting.
2. Until vomiting has ceased no aperient will be retained, nor will remedies for collapse, such as brandy.

Lavage of the stomach may be a good preliminary in some cases, but such measures as emesis provoked by vin. ipecac. are most undesirable. The stomach is usually empty if there has been vomiting, and all it needs is rest. Fasting is the best remedy for vomiting. Should the aperient be vomited, wait again two hours and repeat a similar or a different aperient dose. Thus, if castor oil was the first and was vomited, calomel may be tried next; but in the writer's experience, if the fasting has been strict, it is very rare for castor oil to be rejected a second time. Give nothing whatever by the mouth, neither food nor medicine, till the aperient has been kept down for at least two hours. Write all instructions down on paper, put the paper on the mantelpiece, and catechise the mother at each visit. It is difficult to insist too strongly on the importance of the early emptying and subsequent rest of the stomach and bowels. Unless collapse is markedly present, continue the fast for several hours. Besides being the best remedy for the sickness, fasting is also the best early remedy for the diarrhœa. But if collapse is urgent stimulants are needed, and therefore the experiment must be tried whether the stomach can retain such things as teaspoonfuls either of warm weak brandy and water or champagne and water, but not food of any kind.

The less feeding there is for forty-eight hours the better. If much feeding be persisted in it will probably cause vomiting, which, after all, is perhaps safer than retention, but more wasteful of the child's strength than fasting would be. The child will cry from fasting, but it will cry from pain if it be fed, and it had better cry and get better than cry and get worse. If food be unfortunately given and kept down, it is likely to cause pain from rapid and irregular peristaltic action, and, worse still, it will decompose into a germ nursery for the manufacture of deadly poisons. The hypothesis of poisoning is well supported by the facts that some of the worst cases have not nearly so many bowel actions per diem as some cases that recover; and that some cases, even after the sickness and diarrhœa have diminished or ceased, become comatose and die. The child will not get much benefit from the food, but the bacteria will. The child may get well whilst food is being given, but it is more likely to be in spite of the food than by means of it. Starvation is not the great danger, nor food the great remedy.

We will now suppose that the bowels have been emptied, the vomiting has ceased, the collapse has been energetically treated, and that it is thought time to give a little weak nourishment. White of egg thoroughly well beaten up and then diluted with plenty of nearly cold water is as suitable and more easily to be obtained than anything else. Whey is another excellent food. But for simplicity and readiness the white of egg solution is unrivalled. During the early days of acute infantile diarrhœa begin with a teaspoonful at a time every half-hour or hour, and gradually increase the quantity given at a time. Unless there is vomiting, the supply of water by the mouth need not be limited. If absorbed, it will tend to remedy the collapse by refilling the child's blood-vessels; if not absorbed, it will assist in washing out the bowel.

This matter of keeping the bowels as clear as possible from food residue is of the greatest importance throughout the whole attack. The more pasty and foul the motions are the less food should one give, and the more frequently should aperients, such as castor oil, be given so as to ensure early removal of the waste. Many medical men agree with the use of a preliminary purge, but do not seem to see the need for its repetition. The castor oil is mechanically the most soothing aperient for this purpose, and may be given every day or every other day, a teaspoonful at a time, or two or three times a day a few drops at a time. Small babies seem rather to like it. The white of egg also is a very soothing application. One can hardly conceive of two things less irritating to a sore bowel than castor oil and white of egg solution; in the writer's opinion they, together with the addition, when necessary, of brandy, are for their simplicity, readiness, and efficiency worth more than all the other foods and drugs that he has tried put together. Under this process of having the in-take strictly limited, and of having the waste removed early by castor oil, if necessary, daily, the child wastes as in other methods of treatment, and the crying is not much less, but it is the whining of hunger rather than the cry of pain. The writer has thought that the tongue remains cleaner and moister, and that the temperature does not seem to range so high as in other methods of treatment, nor does he often encounter trouble with vomiting. It certainly seems to him the likeliest way of getting the child over the first week of poisoning.

Breast-fed babies (although subjected to the same chills and heats as the others) do not get diarrhœa so often nor so severely as the artificially fed. The inference is obvious. The writer has not had in his own practice a fatal case of diarrhœa in any baby whose sole food has been the breast. If a breast-fed baby gets diarrhœa, abstinence from the milk is advisable for two to three days in order to be on the safe side. The mother can preserve her future power of supply by the temporary use of a breast-pump.

All cases of infantile diarrhœa must be seen frequently, and the buttocks and stools inspected daily. The baby should never be awakened for food or physic; it may happen that the changing of the napkin may awake it; if so, it cannot be helped, for this must be done. Next, and this is most important, the child should be kept as much as possible in the open air. It will cry less and sleep more under the shade of a tree in a garden or on a balcony at the seaside than in a stuffy room smelling perhaps of vomit or pasty motions; but it must be a private garden, not a public one, or the sympathetic suggestions as to diet given by other mothers or nurses will drive the mother frantic and harass the doctor at his next visit. Whether the practitioner considers diarrhœa to be infectious or not, it will be well for him to make sure that any napkins which are saved for him to see are not kept under the bed, nor anywhere in the child's room, and that they are soon boiled. The writer has not tried irrigation of the colon with one to two pints of saline solution, but very strongly approves of the idea on which it is based, and which he has tried to emphasise—namely, that the cleansing and keeping clean of the bowels should be effectually done. The rectal saline injection, a few ounces at a time for absorption, mentioned previously as one remedy for collapse, is of course a different matter. The writer has used these with benefit. Injections for either rectum or colon may be used cold if the baby is too hot, or fairly hot if the baby is too cold, or just warm if the baby is neither too hot nor too cold. The question of the return to ordinary diet after the attack will not now be discussed; it is important, but the mother often relieves us of all responsibility in that respect by giving milk or other baby food long before we should be disposed to allow it. If such an experiment succeeds, the medical attendant will be informed of it and expected to praise her enterprise; but if a relapse results, he will probably be left to flounder about in search for the cause.

We now come to the question of non-aperient drugs. The writer believes that these are the least important part of the whole treatment. In the first week of acute infantile diarrhœa he gives no medicine to directly check diarrhœa. If it be allowed that the value of aperients is the freeing of the bowels from irritation by waste food-products and the protection of the patient from poisons derived from this source, then all astringents must be bad. Hence, during the first week at any rate, he regards as bad all such drugs as chalk, kino, catechu, logwood, and rhatany. No drugs should be given with the view of reducing twenty motions a day to ten; the number of motions is best controlled by diminishing the amount of irritating matter in the bowels. One would think that the stench of the motions would suggest that they should be removed rather than retained.

Now about opium in these cases. A third of the profession says give it, a third says don't give it, and a third sits on the fence and says that opium needs careful administration. The reason is that some of the effects of opium are good and some are bad. As a nerve soother, allaying pain and fretfulness, diminishing the effects of shock, and procuring rest, opium does good. As an astringent, paralysing the movement of the intestines, it does harm. Moreover, in these cases of acute infantile diarrhœa there is, very occasionally, dropsy, probably from nephritis or renal inadequacy, and opium is then very dangerous. If opium must be given, it is best added to a starch enema; in that way it will not be so astringent to the bowel, and it will be as soothing to the nervous system. The writer prefers not to use it, and seldom finds it needed for pain if the bowels are kept almost empty and the baby is kept warm.

Next with regard to mercurials. Calomel is useful as an aperient if for any reason (such as vomiting) it be preferred to castor oil. A preliminary clearing dose of a grain may be given, and subsequently gr. $\frac{1}{6}$ every few hours for two or three days. Or hyd. cum cret. may similarly be given in a preliminary dose of gr. iij, and subsequently gr. $\frac{1}{3}$ every few hours for two or three days. But these powders have a way of jumping on to the carpet when the paper is opened. Are they better than castor oil? They are less ready. See how moist the lips and tongue are after castor oil. Bismuth is a favourite drug. It seems at any rate to be harmless; it is antiseptic, its astringency cannot be great, and it is a mechanical varnish; ʒij daily may be given. Bicarbonate of soda may be useful for the stomach; but with the acid and alkali question the writer is not competent to deal. He has never tried acids during the first week of infantile diarrhœa. What are we to do when one authority tells us that the familiar green motions are due to a specific bacillus and that acids (such as lactic acid) should be given to kill it; whereas another authority assures us that the popular alkalies (such as soda) are useful for neutralising the irritating organic acids (such as lactic acid) produced by fermentation in the bowels, and that it is these acids which cause diarrhœa by irritating the bowels? Tradition at Guy's says that at the close of several materia medica lectures given by one of our physicians concerning the action of acids and alkalies a student went down to the lecturer's table and quietly asked, "But, sir, when would *you* give acids and when alkalies in stomach troubles?" The reply was, "Mr. W., if you have really followed my lectures as closely as you appear to have done, you will have perceived that I am quite unable to tell you." Of carminatives, brandy is the best. It remains to speak of antiseptics such as corrosive sublimate, salol, salicylates, thymol, resorcin, naphthol, and many others recommended for their germ-killing power.

### Summary.

Diarrhœa is due to something in the diet. Inspect the stools carefully and repeatedly. Empty the bowels and keep them cleansed, preferably by castor oil. Treat collapse energetically, and don't trouble about the number of motions. Don't stop up the bowels by astringents and opiates. Give hardly any food for a few days. Begin with white of egg solution in teaspoonful doses every half hour. If the baby is too hot, cool it; if it is too cold, warm it; if it is thirsty and not sick, give it water; and keep it in the fresh air all day long.

